# *Leishmania (Leishmania) martiniquensis* n. sp. (Kinetoplastida: Trypanosomatidae), description of the parasite responsible for cutaneous leishmaniasis in Martinique Island (French West Indies)

**DOI:** 10.1051/parasite/2014011

**Published:** 2014-03-14

**Authors:** Nicole Desbois, Francine Pratlong, Danièle Quist, Jean-Pierre Dedet

**Affiliations:** 1 CHU de la Martinique, Hôpital Pierre-Zobda-Quitman, Pôle de Biologie de territoire-Pathologie, Unité de Parasitologie-Mycologie BP 632 97261 Fort-de-France Cedex Martinique, France; 2 Université Montpellier 1 et CHRU de Montpellier, Centre National de référence des leishmanioses, UMR « MIVEGEC » (CNRS 5290, IRD 224, UM1, UM2), Département de Parasitologie-Mycologie (Professeur Patrick Bastien) 39 avenue Charles Flahault 34295 Montpellier Cedex 5 France; 3 CHU de la Martinique, Hôpital Pierre-Zobda-Quitman, Service de dermatologie, Pôle de Médecine-Spécialités médicales BP 632 97261 Fort-de-France Cedex Martinique, France

**Keywords:** *Leishmania martiniquensis* n. sp., Cutaneous leishmaniasis, Martinique Island, French West Indies

## Abstract

The parasite responsible for autochthonous cutaneous leishmaniasis in Martinique island (French West Indies) was first isolated in 1995; its taxonomical position was established only in 2002, but it remained unnamed. In the present paper, the authors name this parasite *Leishmania (Leishmania) martiniquensis* Desbois, Pratlong & Dedet n. sp. and describe the type strain of this taxon, including its biological characteristics, biochemical and molecular identification, and pathogenicity. This parasite, clearly distinct from all other *Euleishmania*, and placed at the base of the *Leishmania* phylogenetic tree, is included in the subgenus *Leishmania*.

## Abstract (fr)

Le parasite responsable de la leishmaniose cutanée autochtone de Martinique (Antilles françaises) fut isolé pour la première fois en 1995, mais sa position taxonomique n’a été décrite qu’en 2002, bien qu’il ne soit pas nommé à cette époque. L’objet de cet article est de nommer ce parasite *Leishmania (Leishmania) matiniquensis* Desbois, Pratlong & Dedet n. sp. et de décrire les caractères biologiques, l’identification biochimique et moléculaire et le pouvoir pathogène de la souche type. Ce parasite, distinct de toutes les autres *Euleishmania* et placé à la base de l’arbre phylogénétique des *Leishmania*, est rangé dans le sous-genre *Leishmania.*

## Introduction

Leishmaniasis is a vector-borne parasitic disease caused by Protozoa of the genus *Leishmania*, of worldwide distribution. In the New World, it is endemic from Mexico in the North to Argentina in the South [13]. Sporadic cases have been reported in the Caribbean area, particularly in Trinidad [12] and the Dominican Republic [1]. In Martinique Island, occasional autochthonous cases of cutaneous leishmaniasis (CL)-like infections were reported in 1917 [11] and 1951 [7]. More recently, two new cases were reported with isolation of the parasites, and identified by isoenzyme electrophoresis as monoxenous trypanosomatids [2, 5]. The taxonomical level of the parasites responsible for these two cases (MAR1 and MAR2) was determined by isoenzyme analysis and sequencing of the 18S ribosomal RNA gene, as well as partial sequencing of the DNA polymerase alpha and RNA polymerase II largest subunit genes [10]. This study led these authors to consider these parasites as belonging to the genus *Leishmania*, closely related to the *L. (Leishmania)* subgenus, and located at the base of all other *Euleishmania* [10].

A systematic surveillance of cutaneous lesions in Martinique led to the diagnosis of four new cases between 2004 and 2008 (N. Desbois, unpublished data), the molecular and/or isoenzyme identification of the corresponding parasites showing identity with MAR1 and MAR2.

Therefore, an endemic focus of CL is present in Martinique, due to a well-established *Leishmania* parasite, with a divergent taxonomic position, apart from any other existing taxa. Since the name *Leishmania americana* given by Stevenel in his 1917 paper [11] was lacking a description or a definition, we consider it as a *nomen nudum,* and therefore propose to name this new *Leishmania* taxon and describe its main features.

## *Leishmania (Leishmania) martiniquensis* Desbois, Pratlong & Dedet

urn:lsid:zoobank.org:act:7FB7B26A-3329-44F4-9839-2A3E8AE38324

Class: Kinetoplastea Honigberg, 1963 emend. Vickerman, 1976.

Order: Trypanosomatida Kent, 1880 stat. nov. Hollande 1952

Family: Trypanosomatidae Doflein, 1951

*Authorship*: Note that the authors of the new taxon are different from the authors of this paper; Article 50.1 and Recommendation 50A of the International Code of Zoological Nomenclature [9].

*Strain designation*: MHOM/MQ/92/MAR1.

*Generic assignment*: Amastigotes and promastigotes are the observed morphotypes of this species, which is thereby assigned to the genus *Leishmania* Ross 1903.

Amastigote measurements: diameter: 3.99 ± 0.48 μm.

Promastigote measurements: body length: 9.44 ± 3.02 μm; body width: 2.20 ± 0.63 μm; flagellum length: 11.59 ± 3.63 μm.

*Growth in vitro*:

- Isolation from biopsy material in Schneider’s medium (Serva) supplemented with 10% foetal calf serum, promastigotes obtained in 4–5 days;
- Culture: difficult to grow, doubling time 24 h on SDM79 medium (PAA Laboratories GmbH, Austria) supplemented with 15% foetal calf serum (Invitrogen-Life Technologies, UK – South American origin), 7 μg/mL hemin (Sigma-Aldrich, USA) and 2.5 μg/mL 6-biopterin (Sigma-Aldrich, USA).

*Species assignment*: The new species was identified by molecular and isoenzymatic techniques, showing a parasite clearly distinct from all other *Euleishmania* species and placed at the base of the phylogenetic tree, in the subgenus *Leishmania*.

Molecular typing: GenBank accession numbers: RNA polymerase AF 326982 and DNA polymerase AF 326983 [10], 18S rRNA AF 303938.

Enzyme profiles: MDH^150^, ME^45^, ICD^95^, PGD^87^, G6PD^78^, GLUD^300^, DIA^30^, NP1^00^, NP2^85^, GOT1^170^, GOT2^00^, PGM^104^, FH^65^, MPI^13^,GPI^52^, corresponding to zymodeme MON-229.

*Type host*: man.

Locality in host: skin.

Pathology in man: multiple nodular skin lesions of diffuse cutaneous leishmaniasis pattern, in a patient infected with human immunodeficiency virus (HIV).

Experimental pathogenicity in the murine model: strain infective to BALB/c mice and capable of visceralisation and dissemination in the popliteal and mesenteric lymph nodes, liver, spleen and even brain [8].

*Type locality*: Martinique island (French West Indies).

*Suspected sandfly vector*: Unknown

*Reservoir(s)*: Unkown

*Type material*: Cryopreserved promastigotes were deposited in the Biological Resources Centre of *Leishmania* in Montpellier, France (BRC-Leish, International Cryobank and Identification Centre for *Leishmania*, Hospital University Centre and Université Montpellier 1: http://www.parasitologie.univ-montp1.fr/english_vers/en_cryobanque.htm, and http://www.fbrcmi.fr/?page_id=14&lang=en

*Homologous strains*: Cryostabilates of four strains, belonging to the same zymodeme MON-229, and isolated in various localities of Martinique Island: MHOM/MQ/97/MAR2, MHOM/MQ/2004/MAR7, MHOM/MQ/2007/MAR12 and MHOM/MQ/2008/MAR16

*Etymology*: The species name is a Latin-like word related to the name of the island where this species is endemic and up to now only located.

## Discussion

An endemic focus of cutaneous leishmaniasis has been known in Martinique Island since 1917, but the parasite responsible was clearly identified as a *Leishmania* by molecular and isoenzymatic techniques only in 2002 [10]; the taxonomic position of this parasite has already been described: it was clearly distinct from all other *Euleishmania*, placed at the base of the phylogenetic tree, in the subgenus *Leishmania*. We therefore propose to name this parasite *Leishmania (Leishmania) martiniquensis*, Desbois, Pratlong & Dedet n. sp.

Since the isolation of the type strain in 1992, the four other strains isolated caused cutaneous lesions, the clinical description of which can be summarised as follows: single lesion of the face, of papulo-nodular type, with or without ulceration, sometimes mimicking a basocellular carcinoma. The lesions were localised to the periorbital area, one to the forehead and the last on the ear lobe. Their evolution was spontaneously favourable over several months.

Apart from the princeps case of 1992, which caused a diffuse CL in an immunocompromised patient, the four other cases corresponded to localised CL in immunocompetent patients. The clinical polymorphism of the localised lesions in immunocompetent patients, and the diffuse lesions in the immunocompromised one are not specific to this new species, but similar to clinical features of CL due to other *Leishmania* species [4].

From an epidemiological point of view, the *L. martiniquensis* focus appears to be endemic in Martinique Island, with occurrence of rare human cases. The existence of an animal reservoir is highly likely, the candidate reservoirs being one of the wild rodents or marsupials present in Martinique, such as black rats (found infected in Costa Rica by Zeledon in 1992 [14]) or opossums (reservoir hosts in French Guiana [3]). Recent entomological surveys carried out in Martinique Island confirmed the presence of *Lutzomyia atroclavata*, isolated for the first time in 1966 in Martinique [6] and reported the presence of a second phlebotomine sandfly species: *Lutzomyia cayennensis* (Desbois N., unpublished).

## Conclusion

At the time of writing, the parasite presently described has never been found outside of Martinique Island. The geographical isolation of this parasite in Martinique might explain its maintenance as an ancestral *Leishmania*. It remains to determine its vector(s) and reservoir host(s), and to explain why this parasite has not extended to neighbouring countries in the Caribbean area.
